# A High-Density Genetic Map Enables Genome Synteny and QTL Mapping of Vegetative Growth and Leaf Traits in Gardenia

**DOI:** 10.3389/fgene.2021.802738

**Published:** 2022-01-04

**Authors:** Yang Cui, Baolian Fan, Xu Xu, Shasha Sheng, Yuhui Xu, Xiaoyun Wang

**Affiliations:** ^1^ Research Center for Traditional Chinese Medicine Resources and Ethnic Minority Medicine, Jiangxi University of Chinese Medicine, Nanchang, China; ^2^ Adsen Biotechnology Co., Ltd., Urumchi, China

**Keywords:** genetic map, genotyping-by-sequencing, growth-and leaf-related traits, QTL, synteny, gardenia

## Abstract

The gardenia is a traditional medicinal horticultural plant in China, but its molecular genetic research has been largely hysteretic. Here, we constructed an F_1_ population with 200 true hybrid individuals. Using the genotyping-by-sequencing method, a high-density sex-average genetic map was generated that contained 4,249 SNPs with a total length of 1956.28 cM and an average genetic distance of 0.46 cM. We developed 17 SNP-based Kompetitive Allele-Specific PCR markers and found that 15 SNPs were successfully genotyped, of which 13 single-nucleotide polymorphism genotypings of 96 F_1_ individuals showed genotypes consistent with GBS-mined genotypes. A genomic collinearity analysis between gardenia and the *Rubiaceae* species *Coffea arabica*, *Coffea canephora* and *Ophiorrhiza pumila* showed the relativity strong conservation of LG11 with NC_039,919.1, HG974438.1 and Bliw01000011.1, respectively. Lastly, a quantitative trait loci analysis at three phenotyping time points (2019, 2020, and 2021) yielded 18 QTLs for growth-related traits and 31 QTLs for leaf-related traits, of which *qBSBN7-1*, *qCD8* and *qLNP2-1* could be repeatably detected. Five QTL regions (*qCD8* and *qSBD8*, *qBSBN7* and *qSI7*, *qCD4-1* and *qLLLS4*, *qLNP10* and *qSLWS10-2*, *qSBD10* and *qLLLS10*) with potential pleiotropic effects were also observed. This study provides novel insight into molecular genetic research and could be helpful for further gene cloning and marker-assisted selection for early growth and development traits in the gardenia.

## Introduction

Gardenia (*Gardenia jasminoides* Ellis, 2n = 22) originated in central China, and it is a perennial shrub in the Rubiaceae family with edible flowers and medicinal fruits. Its dried ripe fruit has high quantities of crocin, geniposide, and genipin compounds (Chen Q. et al., 2020) and therefore possesses anti-inflammatory, antidepressant, anti-diabetes, antioxidant and antihypertensive activities (Qin et al., 2013; Higashino et al., 2014; Khajeh et al., 2020). The fruits are used in many traditional Chinese medicine preparations and formulas to treat different diseases (Chen L. et al., 2020). In addition to applications in traditional Chinese medicine, extracts of gardenia fruit are used as a natural colorant in the food and textile industries (Chen L. et al., 2020). Gardenia has beautiful fragrant flowers and evergreen leaves, so it is widely used for garden decoration. Fresh flowers are also used in China as edible vegetables or used to extract essential oils (Wang et al., 2017). Gardenia has a cultivation history of more than 1,000 years in China and was gradually introduced to Africa, Asia, Australia, Europe, North and South America, and the Pacific islands because of its medicinal, ornamental and industrial value (Xu et al., 2020).

Using traditional phenotypic selection-based breeding methods for genetic improvement is a labor- and time-consuming process because of the long lifecycle and highly heterozygous nature of the gardenia. By contrast, marker-assisted selection (MAS) using tightly linked or functional molecular markers with elite traits is an ideal approach to improving breeding efficiency (Mathew et al., 2014; Pootakham et al., 2015; Dong et al., 2019). However, the current molecular biology research for gardenia falls further behind model species, primarily focusing on phenotype, genetic evaluation or accession discrimination (Tsanakas et al., 2013; Hu et al., 2019; Wei et al., 2019; Li et al., 2021). Very limited studies on molecular marker identification in gardenia have been reported, such as dozens of SSR developments (Xu et al., 2014; Deng et al., 2015). Recently, a chromosomal-level genome assembly for the gardenia was released to dissect the pathway of crocin biosynthesis (Xu et al., 2020). Furthermore, helix-loop-helix (bHLH) transcription factors responsible for crocin biosynthesis were identified based on the gardenia genome (Tian et al., 2020). Genome assembly will undoubtedly accelerate functional genomics studies in gardenia. Nevertheless, the notably shortage of genome-wide molecular marker and the large gap between the phenotyping and genotyping are still bottlenecks for gardenia genetic improvement by molecular breeding and thus restrict the gardenia related industry.

Genetic maps based on the F_1_ segregating population are a robust tool for identifying the linkage between traits and molecular markers, which have long been applied widely in highly heterozygous species of trees, flowering plants and aquatics (Jorge et al., 2005; Wang et al., 2006; Lambert et al., 2007; Oyant et al., 2007; Sánchez-Pérez et al., 2012; Pacheco et al., 2014). In the next-generation sequencing (NGS) era, sequencing-based technologies can provide novel strategies for genome-wide SNP (single-nucleotide polymorphism) development and help to construct a high-density genetic linkage map for high-resolution QTL (quantitative trait loci) identification (Rehman et al., 2020). SNP markers can be named in many ways, including reduced-representation sequencing, resequencing and transcriptome sequencing. Reduced-representation sequencing has been differentiated into different technologies, including genotyping-by-sequencing (GBS), restriction site-associated DNA sequencing (RAD-Seq), double-digest RAD (ddRAD), specific-locus amplified fragment sequencing (SLAF-seq), ezRAD (Toonen et al., 2013) and 2b-restriction site-associated DNA sequencing (2b-RAD) (Baird et al., 2008; Elshire et al., 2011; Peterson et al., 2012; Wang et al., 2012; Sun et al., 2013; Toonen et al., 2013). Notably, GBS is a feasible SNP discovery method for highly diverse and large genome species, even without reference genome, and it has been widely adopted in genotyping for genetic map construction (İpek et al., 2017; Gabay et al., 2018; Paudel et al., 2018; Robledo et al., 2018; Lewter et al., 2019; Rubio et al., 2020). For instance, a high-density linkage map of coffee, a tree belongs to the same *Rubiaceae* family with gardenia, was constructed using 848 SSR and SNP markers, of which the SNP markers were developed by GBS (Moncada et al., 2016). Additional high-density genetic maps with 3,000–6,000 SNP markers have been reported in many perennial plants (Pootakham et al., 2015; Zhang et al., 2016; İpek et al., 2016; Tello et al., 2019; Zhang et al., 2019). GBS was also used for genetic diversity analysis in coffee (Anagbogu et al., 2019).

Genetic map can provide chromosome-level variation information across species. In fact, the microsynteny and macrosynteny relationship have long been verified in plants (Paterson et al., 2004; Yan et al., 2004). Comparative mapping can illustrate the co-located molecular marker distribution patterns between different genome of organisms, and further reveal structural variations and collinearity among chromosomes. Using this method, a high degree of colinearity and chromosome recombination and inversion has been found in *Salicaceae* species (Hanley et al., 2006; Berlin et al., 2010). Similarly, chromosomal translocations and inversions were confirmed by comparing an eggplant genetic map with the genome sequence of both tomato and pepper (Rinaldi et al., 2016). Lately, the genomic evolutionary of *Coffea canephora* and *Ophiorrhiza pumila* were investigated (Zhao et al., 2021), and some high collinearity pairs and potential karyotype rearrangement were observed, indicating their chromosomal evolution in genomic differentiation (Kai et al., 2011; Kodama et al., 2014).

QTL mapping is a traditional method to build an association bridge between genotypes and phenotypes. The tightly linked markers in QTL regions can potentially be used for MAS (Chang et al., 2018; Kim et al., 2018; Yamakawa et al., 2021). The phenotypes for typical QTL mapping always focus on specific developmental stages, and the identified QTLs represent the accumulation effect of related gene expression at the phenotyping stages. However, plant growth and development are dynamic, ever-changing processes. Dynamic QTL analysis enables QTL detection for target traits over the entire developmental process, especially for tree species, which require a relatively long time for morphogenesis. Dynamic QTL mapping studies have been published primarily for crops such as rice (Sun et al., 2015), maize (Wang et al., 2019), wheat (Mohler and Stadlmeier, 2019), cotton (Shang et al., 2015) and oilseed rape (Wang et al., 2015). In tree species, however, limited dynamic QTL maps were conducted. Desnoues et al. (2016) reported the dynamic QTL mapping of fresh weight, sugar, acid and enzyme activity at different developmental stages of peach fruit, and observed the effect of allele changes during fruit ripening. Recently, the leaf traits and plant height of *Catalpa bungei* at five successive time points were investigated, and a total of 33 QTLs were mapped using a high-density genetic map (Lu et al., 2019). In *Populus*, a total of 311 QTLs for three growth traits at 12 time points were mapped, and many QTLs specific to one time point were identified (Du et al., 2019). These results illustrated the importance of dynamic QTL mapping for the genetic dissection of developmental traits.

The genetic map of the gardenia has not been released to date. In the present study, we used a paternity test-passed F_1_ population of gardenia, and then employed GBS technology to construct a high-density genetic map for collinearity analysis between *Rubiaceae* species. Moreover, a high-resolution dynamic QTL mapping analysis was performed on growth and leaf related traits during the vegetative growth stage for three continuous years. This study was the first high-density genetic map-based QTL study in gardenia, laying a foundation for further gene cloning and MAS breeding.

## Materials and Methods

### Mapping Population Construction and Phenotyping

We previously screened two *Gardenia jasminoides* Ellis. germplasms that exhibited distinct phenotypes, namely, GD1 with high branches, large fruit, medium leaf widths and a broad crown type and AX5 with dwarf branches, small fruit, narrow leaf widths and a thin crown type. In May 2017, following emasculation at the early stage of flower development, GD1 (♀) and AX5 (♂) were crossed by artificial pollination. The dark red fruits were harvested during the first frost. The hybrid seeds were isolated and then placed on moist germination paper in Petri dishes in November 2017. At the time of radicle protrusion, the seeds were transferred into pots in the greenhouse. During the following year on March 27, the seedlings were transplanted within the Botanic Garden at Jiangxi University of Chinese Medicine (N28N°40′, E115°45′). The two parents and a total of 207 F_1_ individuals were randomly planted. In April 2019, young leaves from the two parents and all the F_1_ individuals were harvested and stored in a silica-gel drier for further DNA extraction.

### Phenotyping and Data Processing

**FIGURE 1:**
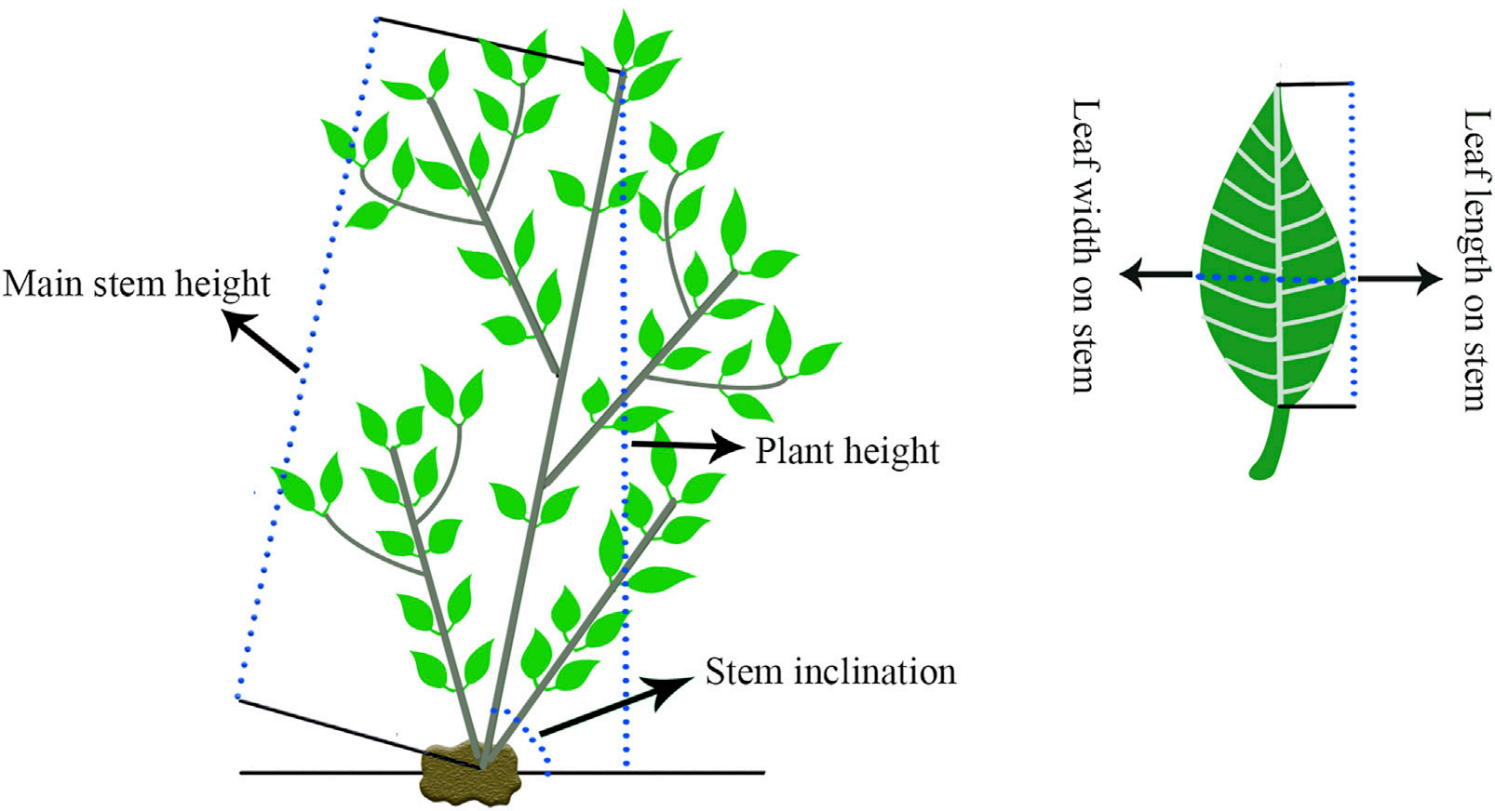
Part of the trait measurement schematic diagram.

We measured 12 traits over three continuous years in October 2019, July 2020 and April 2021, and all the traits were measured three times. The detailed measurements are shown in Table 1 and Figure 1. Protractors and Vernier calipers were used to measure the stem inclinations and stem base diameters, respectively. The flexible rule was used to measure the remaining traits. SPSS V17.0 software (SPSS Inc., Chicago, IL, United States) was used for variance analysis. TBtools was used to display the variation and Pearson pairwise correlations graphically among different traits (Chen C. et al., 2020).

**TABLE 1:** Detailed measurement methods for the 12 agronomic traits.

Trait	Abbreviation	Description
Crown diameter	CD	Measuring the diameter of the identifiable three-dimensional cylinder of each individual tree
Basal stem branch number	BSBN	Counting the branch numbers derived from the basal stem
Stem inclination	SI	See Figure 1
Plant height	PH	See Figure 1
Main stem height	MSH	See Figure 1
Stem base diameter	SBD	Diameter of the stem base
Leaf number on stem	LNS	Counting all the leaf numbers on the main stem
Leaf number per plant	LNP	Counting all the leaf numbers per plant
Longest leaf length on stem	LLLS	Length of the longest leaf on the stem (Figure 1)
Longest leaf width on stem	LLWS	Width of the longest leaf on the stem (Figure 1)
Shortest leaf length on stem	SLLS	Length of the shortest leaf on the stem (Figure 1)
Shortest leaf width on stem	SLWS	Width of the shortest leaf on the stem (Figure 1)

### Paternity Test

To ensure an expectant hybrid seed panel, a paternity test was conducted using simple sequence repeat (SSR) markers. The total DNA from the two parents and 207 F_1_ individuals was isolated separately using DNA Rapid Extraction Kit DN1403 (Aidlab Biotechnologies Co., Ltd., Beijing, China). Using the total DNA from the two parents, a total of 25 SSRs from Deng et al. (2015) were used for polymorphic screening. Homozygous and polymorphic SSR markers were selected to genotype the 207 F_1_ individuals. For example, if the genotypes of the two parents were encoded with “aa” and “bb”, then the genotype of the true F_1_ offspring was “ab”. The polymerase chain reaction (PCR) system for SSR genotyping was performed in a 10.0 μl volume, with 5 μl 2×Taq MasterMix, 0.2 μl forward primer (F) and reverse primer (R), respectively 1 μl sample genomic DNA and 3.6 μl ddH_2_O. The system was pre-degenerated at 94°C for 3 min, and then PCR amplification began for 34 cycles of 94°C for 30 s, 55°C for 30 s, 72°C for 30 s, and a final extension at 72°C for 5 min. An 8% denaturing polyacrylamide gel was used to separate the PCR products for further silver staining.

### Population GBS Sequencing and Genotyping

Similar to the paternity test, genomic DNA was isolated from the young leaves of GD1, AX5 and 200 true hybrid F_1_ individuals using the DNA Rapid Extraction Kit DN1403 (Aidlab Biotechnologies Co., Ltd., Beijing, China). The DNA concentration and quality were monitored using a NanoDrop spectrophotometer (ND 2000, Thermo Fisher Scientific, United States) and electrophoresis on 0.85% agarose gels, respectively. Then, GBS libraries were constructed. In brief, the genomic DNA was placed into a combination solution of *RsaI* and *HaeIII* for digestion. Products between 429 and 459 bp in length were enriched in 3% agarose gels, and end repair was performed with End Prep Enzyme Mix, followed by 3′A extension and adaptor addition. The dual index for further sample identification was introduced by PCR with eight cycles. Library quantification was performed using an Agilent 2,100 Bioanalyzer (Agilent Technologies, Palo Alto, CA, United States), and all the libraries were mixed into one lane for paired-end sequencing (PE150) at Adsen Biotechnology Co., Ltd (Urumchi, China) using an Illumina NovaSeq 6,000 (Illumina, San Diego, CA, United States). The raw data were filtered to generate high-quality clean data according to Zhao’s criteria (Zhao et al., 2021). The genotyping was processed according to the following steps. First, a Burrows-Wheeler aligner (Li and Durbin, 2009) was used to map the clean reads to the reference genome of gardenia (Xu et al., 2020), followed by duplicate removal (Picard: http://sourceforge.net/projects/picard/). Second, SNPs were called by combining the HaplotypeCaller module of GATK (McKenna et al., 2010) and SAMtools (Li et al., 2009) to guarantee a high-quality SNP dataset. Lastly, dual-detected SNPs with sequencing depths ≥8 in the two parents, segregation distortion *p* > 0.01 (Chi-square) and integrity ≥60% in the offspring were maintained and encoded into eight genotyping patterns suitable for diploid species (aa × bb, ab × cd, ef ×, e.g., hk × hk, lm × ll, nn × np, ab × cc, and cc × ab). All the genotypes except aa × bb were selected and SMOOTH algorithms (van Os et al., 2005) were used to correct genotypes and imputation for further genetic map construction.

### Genetic Linkage Map Construction, QTL Mapping and Gene Annotation Analyses

All the retained SNP markers were assigned into linkage groups (LGs) based on the mapping location on the reference genome of the gardenia (Xu et al., 2020). JoinMap software (V4.1) was applied for linear arrangement within LGs using the mapping function of the cross pollination (CP) model (Van Ooijen 2006). Map distances were estimated using the Kosambi mapping function (Kosambi, 1943). Genetic map visualization was performed using a CheckMatrix heat plot (http://cgpdb.ucdavis.edu/XLinkage/). The Spearman correlation coefficient between the final LGs and the reference genome was calculated and visualized using R (www.r-project.org/). MapQTL V6.0 was used for QTL analyses using the interval mapping (IM) algorithm (Van Ooijen 2009). QTLs were cut off when the LOD (logarithm of odds) values of three continuous SNPs were ≥2.5. Genes underlying stable expressed QTLs were annotated by ANNOVAR (Wang et al., 2010), and functional enrichment analyses were conducted by UniProtKB/Swiss-Prot database (Schneider et al., 2004), Pfam (Bateman et al., 2004), Gene Ontology (Ashburner et al., 2000) and KEGG (kyoto encyclopedia of genes and genomes) (Kanehisa and Goto, 2000).

### Genome Synteny Analyses

To explore the evolutionary relationship between gardenia and other *Rubiaceae* species with chromosome-level genomes, SNP-based high-density genetic maps were aligned to the genomes of *Coffea canephora* (https://www.ncbi.nlm.nih.gov/genome/12248), *Coffea arabica* (https://www.ncbi.nlm.nih.gov/genome/?term=Coffea+arabica) and *Ophiorrhiza pumila* (https://www.ncbi.nlm.nih.gov/genome/97777?genome_assembly_id=1538555) using BLAST (Kent, 2002), and the physical positions of the homologous sequences were used to generate a collinearity diagram in R (www.r-project.org/).

### SNP Confirmation by Kompetitive Allele-Specific PCR (KASP)

To confirm the SNPs developed by GBS, we randomly genotyped 96 F_1_ individuals by KASP using 17 SNPs from five randomly selected QTL regions (Supplementary Table S1). Primer 5.0 was used to design the primers, and BLAST (https://blast.ncbi.nlm.nih.gov/Blast.cgi?PROGRAM=blastn&PAGE_TYPE=BlastSearch&LINK_LOC=blasthome) was used to check the primer specificity. The primer information is shown in Supplementary Table S1. The KASP genotyping processes were conducted in the GeneMatrix system (HC Scientific, Chengdu, China) according to the following three parts: Matrix Arrayer reaction plate preparation apparatus, Matrix Cycler high-throughput water bath thermal cycler, and Matrix Scanner high-speed fluorescence scanner. The PCR system contained 1 μl 2×KASP Master mix (standard ROX) (LGC Biosearch Technologies, United KIngdom), 0.028 μl KASP primer mix and 1 μl sample DNA (∼50 ng/μl). The detailed KASP thermal cycling program was 94°C for 15 min, followed by 10 cycles of 94°C for 20 s, 61–55°C for 20 s (dropping 0.6°C per cycle), 72°C for 45 s, 30 cycles of 94°C for 20 s, and 55°C for 1 min.

## Results

### Hybridization Test

In the present study, the parents GD1 and AX5 were used as materials, and 47 published SSRs were used for polymorphism tests. A total of 19 pairs of primers were observed to be polymorphic. We further selected markers that were homozygous and polymorphic in the parents. That is, aa×bb-type polymorphic SSRs eGJ026 and eGJ118 were used for genotyping the progeny. A total of 200 progeny out of 207 individuals in the F_1_ population were true hybrids with the segregation type “ab” (Supplementary Table S1–S2), indicating that the hybridization experiment was strictly controlled.

### Genetic Variations in 12 Phenotypes

**FIGURE 2:**
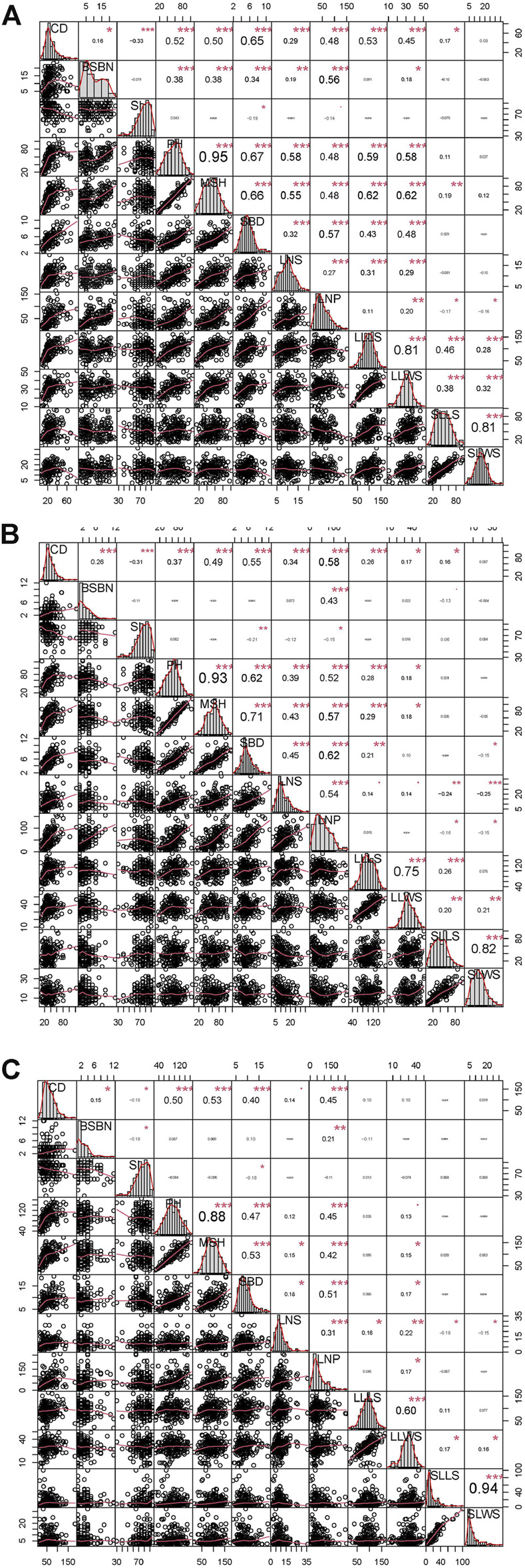
Variation and Pearson pairwise correlation analyses of growth-related and leaf-related traits of the F_1_ population. **(A)**, **(B)** and **(C)** represent the variation and Pearson pairwise correlations in 2019, 2020 and 2021, respectively. The correlations were calculated with Spearman correlation coefficients, and the *p* values are indicated as follows: *, *p* < 0.05; **, *p* < 0.01; and ***, *p* < 0.001. The abbreviations given in the histograms are as follows: CD: crown diameter; BSBN: basal stem branch number; SI: stem inclination; PH: plant height; MSH: main stem height; SBD: stem base diameter; LNS: leaf number on stem; LNP: leaf number per plant; LLLS: longest leaf length on stem; LLWS: longest leaf width on stem; SLLS: shortest leaf length on stem; and SLWS: shortest leaf width on stem.

A set of relatively wide ranges of variations were observed in the crown diameter (CD), stem inclination (SI), plant height (PH), main stem height (MSH), leaf number per plant (LNP), the longest leaf length on stem (LLLS) and the shortest leaf length on stem (SLLS), while mild variations were present in the remaining five phenotypes (Supplementary Table S2). The coefficient of variation (CV) of the leaf-related trait LLLS and the longest leaf width on stem (LLWS) were primarily stationary at the three time points, which was similar to all six growth-related traits, suggesting minor differences among these three time points. Only the leaf-related traits LNS, LNP, SLLS and SLWS demonstrated acute CV fluctuation (Supplementary Table S2). Among every year for the phenotypes at the three time point, CD, PH and LNP exhibited strong correlations with other phenotypes. However, SI had no highly significant correlations with the other ten traits (except SBD). PH had the strongest positive correlations with MSH, with correlation coefficients of 0.95, 0.93 and 0.88 in 2019, 2020 and 2021, respectively. SLLS and SLWS also exhibited a correlation greater than 0.80 in the 3 years (Figure 2). The correlation analysis implied that there was an independent and interdependent relationship between growth-related traits and leaf-related traits.

### Variation Calling and Genotyping

In total, GBS sequencing generated 29, 630, 679 clean reads after quality control, with 1.77 and 0.83 Gb for the parent AX5 and GD1, respectively. For the offspring, 12, 186, 237 reads (1.81 Gb) were obtained per individual. The statistics showed that the average Q30 was higher than 85%, and the GC content (%) was distributed between 40.29 and 48.18 (Supplementary Table S3). Upon using BWA software to align the sequencing data to the reference genome of gardenia, the mapping rates were 94.38, 93.88 and 96.66% for AX5, GD1 and all the progeny, respectively. These pre-processing procedures indicated a high quality of sequencing data for further analysis.

**FIGURE 3:**
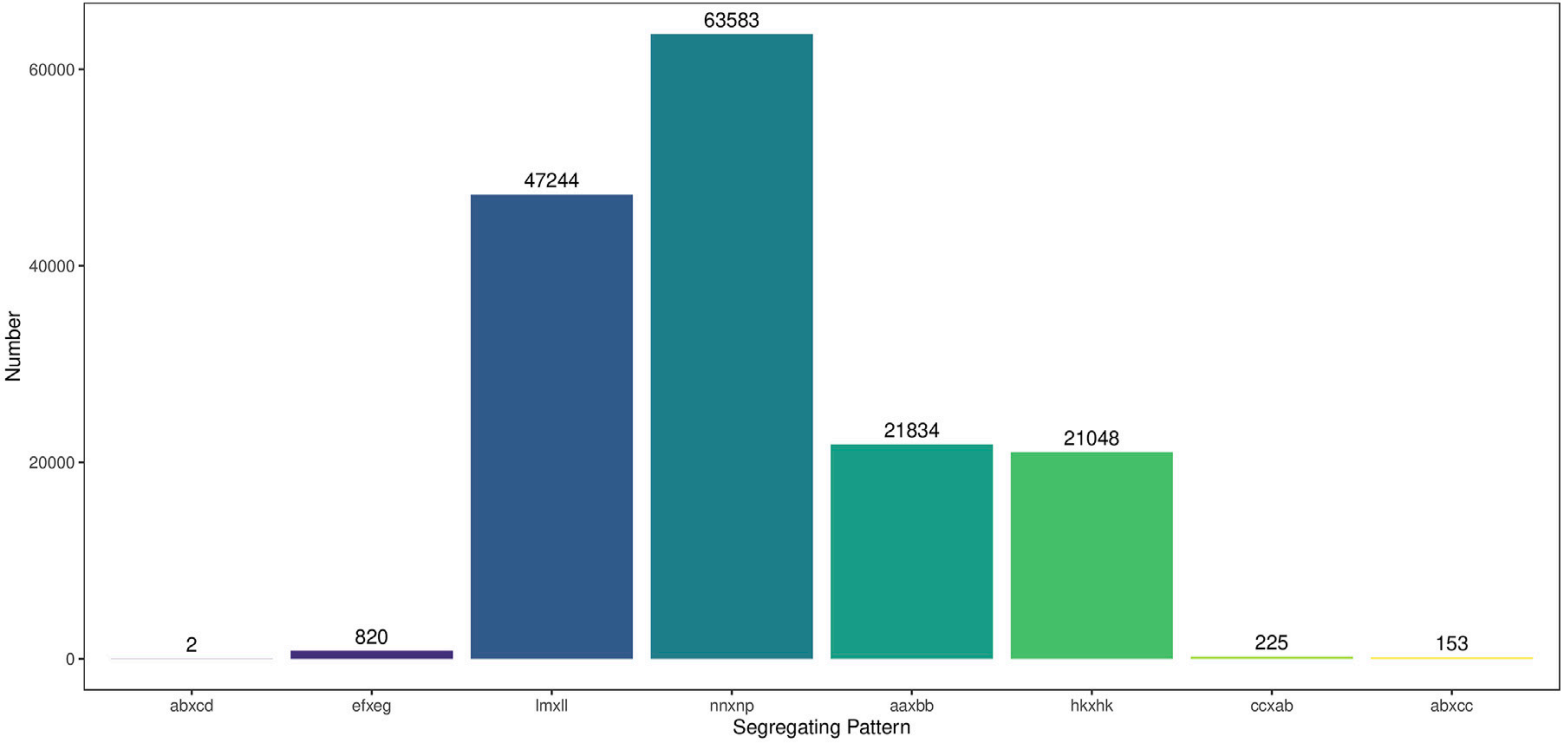
The distributions of SNP marker segregation patterns.

A total of 154,909 SNPs were detected by combining the GATK and SAMTools, and the genotypes of these SNPs were encoded into eight segregation patterns. Among them, lm×ll, np×nn, hk×hk and aa×bb occupied 47,244, 63,583, 21,048 and 21,834 SNPs, respectively, accounting for 99.23% of the total SNPs (Figure 3). After depth and integrity filtering, the remaining SNPs were used for genetic map construction.

### High Density Genetic Map

**FIGURE 4:**
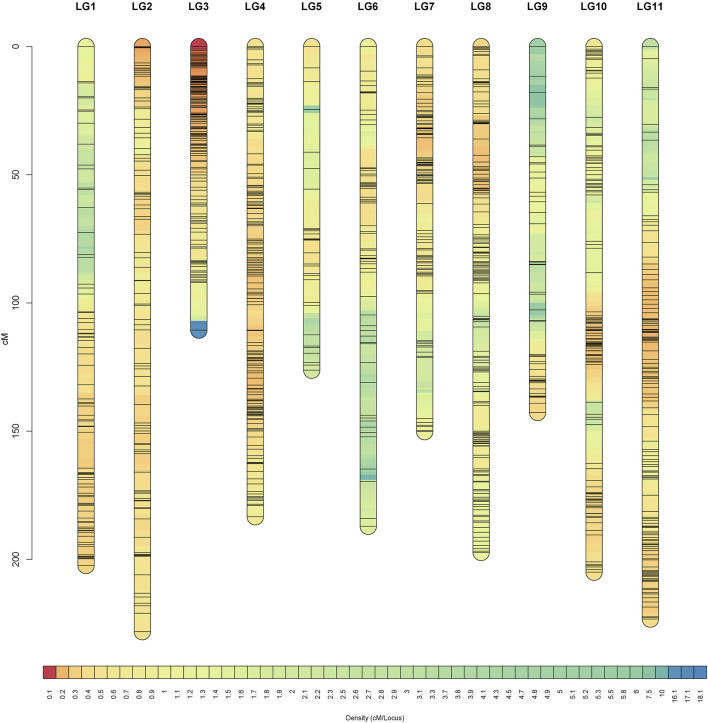
High-density sex-average genetic map of gardenia.

JoinMap was used to construct a female genetic map containing 2,585 markers spanning 2,348.48 cM and a male genetic map containing 1,963 markers spanning 1,348.38 cM, and both consisted of 11 LGs (Table 2). Integrating the female and male maps formed a sex-average genetic map, which included 4,249 SNPs with a total length of 1956.28 cM and an average genetic distance of 0.46 cM (Table 2; Figure 4). Among them, LG2 was the longest (228.19 cM), including 648 SNPs, and the average genetic distance was 0.35 cM. Conversely, LG3 was the shortest group (110.56 cM) with 468 SNP tags but a higher resolution of 0.24 cM between adjacent markers on average. There were 120 SNPs in LG9, which was the least in all 11 LGs, with 1.19 cM, the largest average distance between adjacent markers. Of the 11 LGs, the proportions of genetic gaps (≤ 5 cM) ranged from 92.44 to 99.79%, with 96.86% on average. No genetic gaps larger than 10 cM were observed in LG2, LG4 or LG8. The largest gap presented on LG7, which was 23.86 cM (Table 2). The detailed information of all the SNPs and the corresponding genetic and physical positions were displayed in Supplementary Table S4.

**TABLE 2:** The basic characteristics of the female genetic map, male genetic map and sex-average genetic map.

LG	Marker number			Gap≤ 5 cM (%)			Max gap (cM)			Total distance (cM)			Average distance (cM)		
	Sex-average	Female	Male	Sex-average	Female	Male	Sex-average	Female	Male	Sex-average	Female	Male	Sex-average	Female	Male
1	403	322	100	96.77	95.02	93.94	14.08	23.9	30.2	202.35	217.7	114.49	0.5	0.68	1.14
2	648	352	348	97.37	92.31	97.69	7.19	17.48	13.72	228.19	376.23	80.16	0.35	1.07	0.23
3	468	242	272	99.79	98.76	99.63	18.52	24.71	15.06	110.56	131.34	68.76	0.24	0.54	0.25
4	606	456	183	99.34	98.46	95.05	9.91	8.72	17.83	183.36	174.16	167.7	0.3	0.38	0.92
5	136	62	77	92.59	91.8	89.47	16.59	67.35	10.64	126.19	152.99	87.51	0.93	2.47	1.14
6	194	119	85	93.78	89.83	96.43	16.83	60.2	16.42	187.07	303.73	67.31	0.96	2.55	0.79
7	312	157	170	98.07	98.72	97.63	23.86	25.72	48.88	150.18	104.37	156.02	0.48	0.66	0.92
8	369	187	210	98.37	91.94	96.65	9.96	17.46	15.06	197.3	249.66	144.94	0.53	1.34	0.69
9	120	81	52	92.44	91.25	82.35	14.72	44.89	23.37	142.71	137.78	144.08	1.19	1.7	2.77
10	406	245	189	98.27	95.9	96.81	18.08	29.89	33.67	205.04	244.18	143.14	0.51	1	0.76
11	587	362	277	98.63	96.68	97.46	17.49	17.83	31.74	223.33	256.34	174.27	0.38	0.71	0.63
Total	4,249	2,585	1,963	96.86	94.61	94.83	23.86	67.35	48.88	1956.28	2,348.48	1,348.3	0.46	0.91	0.69

The Spearman correlation coefficient between the genetic map and the reference genome was approximately 0.722 and up to 0.901, indicating that the marker ordering of the genetic map was basically accurate (Supplementary Table S5).

### QTLs for Growth and Leaf-Related Traits

We divided the 12 traits into two categories, namely, phenotypes associated with gardenia growth (CD, BSBN, SI, PH, MSH, and SBD) and leaves (LNS, LNP, LLLS, LLWS, SLLS, and SLWS). Using the high-density genetic map and continuous phenotypic data, 23, 22, and 9 QTLs were mapped in 2019, 2020 and 2021, respectively, of which 18 QTLs were associated with the growth traits, while 31 QTLs were related to the leaf-related traits (Table 3). These QTLs were distributed in all the LGs of gardenia except LG6, with phenotypic variance explained (PVE) values ranging from 5.6 to 11.7%.

**TABLE 3:** QTL mapping results.

Year	QTL	LG	Map position		Supporting SNPs	LOD	PVE (%)
			Start (cM)	End (cM)			
2021	qCD8	8	182.843	183.257	3	3.26–3.35	8.80–9.00
2021	qSBD8	8	182.843	183.257	3	3.17–3.31	7.90–8.20
2021	qBSBN7-1	7	119.234	121.26	5	4.29–4.34	10.00–10.10
2021	qBSBN7-2	7	145.122	146.645	3	4.13–4.29	9.60–10.00
2021	qLNS9	9	120.123	131.097	29	3.27–3.65	7.70–8.60
2021	qLNP1	1	92.668	96.534	4	2.88–2.97	6.80–7.00
2021	qLNP2-1	2	19.951	21.234	6	2.65–2.65	6.30–6.30
2021	qLLWS9	9	69.094	85.132	11	3.02–3.36	7.10–7.90
2021	qSLLS3	3	77.053	78.566	5	2.60–2.64	6.20–6.30
2020	qCD8	8	182.843	183.257	3	4.11–4.24	9.20–9.50
2020	qBSBN7-1	7	119.234	148.157	11	3.15–3.58	7.10–8.10
2020	qSI7	7	119.234	146.645	9	2.60–2.83	5.90–6.40
2020	qSI4-1	4	78.288	83.568	16	2.65–2.95	6.00–6.70
2020	qSBD10	10	190.469	202.015	11	3.20–3.41	7.20–7.70
2020	qLNS7	7	85.434	86.947	5	3.05–3.30	6.90–7.50
2020	qLNP2-1	2	19.951	21.234	6	2.72–2.73	6.20–6.20
2020	qLNP2-2	2	56.792	56.792	4	2.56–2.56	5.80–5.80
2020	qLNP2-3	2	61.8	63.34	7	2.51–2.60	5.70–5.90
2020	qLNP2-4	2	67.325	67.325	4	2.55–2.55	5.80–5.80
2020	qLNP2-5	2	82.433	82.433	5	2.50–2.50	5.70–5.70
2020	qLNP2-6	2	100.356	100.94	16	2.50–2.61	5.70–5.90
2020	qLNP2-7	2	123.624	123.624	3	2.55–2.55	5.80–5.80
2020	qLNP2-8	2	146.796	147.817	11	2.50–2.55	5.70–5.80
2020	qLNP2-9	2	197.261	197.261	15	2.55–2.55	5.80–5.80
2020	qLNP7-1	7	81.885	83.9	9	2.52–2.65	5.70–6.00
2020	qLNP7-2	7	95.235	115.701	11	2.75–2.94	6.30–6.70
2020	qLLLS9	9	69.094	69.094	3	5.30–5.30	11.70–11.70
2020	qSLLS11-1	11	211.278	215.045	27	2.50–2.58	5.70–5.90
2020	qSLLS11-2	11	218.617	223.331	11	2.66–2.72	6.10–6.20
2020	qSLLS5	5	70.972	71.265	3	2.67–2.78	6.10–6.30
2020	qSLWS10	10	183.656	185.741	4	3.10–3.22	7.00–7.30
2019	qCD11-1	11	207.47	208.655	13	2.57–2.57	5.80–5.80
2019	qCD11-2	11	211.779	213.514	15	2.55–2.55	5.70–5.70
2019	qCD4-1	4	98.185	110.8	31	2.53–2.91	5.70–6.50
2019	qLLLS4	4	100.7	100.7	5	3.79–3.79	8.40–8.40
2019	qCD4-2	4	116.649	116.649	6	2.51–2.51	5.60–5.60
2019	qCD4-3	4	119.049	119.632	8	2.53–2.57	5.70–5.80
2019	qCD4-4	4	123.824	126.524	15	2.50–2.53	5.60–5.70
2019	qCD4-5	4	137.479	138.232	7	2.50–2.97	5.60–6.60
2019	qCD4-6	4	143.373	144.048	6	2.56–2.65	5.70–6.00
2019	qCD8	8	182.843	184.973	3	2.51–3.00	5.60–6.70
2019	qSI4-2	4	75.123	76.966	12	4.23–4.40	9.30–9.70
2019	qMSH7	7	88.459	89.464	9	2.50–2.77	5.60–6.20
2019	qSBD11	11	36.459	36.459	7	2.54–2.54	5.70–5.70
2019	qLNS8	8	118.779	122.308	4	3.21–3.27	7.20–7.30
2019	qLNP10	10	12.269	12.269	5	3.05–3.05	6.80–6.80
2019	qSLWS10-2	10	10.417	12.269	6	2.72–2.72	6.10–6.10
2019	qLNP2-1	2	19.951	28.282	15	3.06–3.42	6.80–7.60
2019	qLNP2-10	2	57.294	58.434	17	3.05–3.09	6.80–6.90
2019	qLLLS10	10	202.015	202.015	3	3.71–3.71	8.20–8.20
2019	qLLLS11	11	203.027	205.106	7	3.70–3.76	8.20–8.30
2019	qLLWS4	4	115.455	116.649	5	2.64–2.66	5.90–6.00
2019	qSLLS11-3	11	222.728	222.728	3	3.83–3.83	8.50–8.50
2019	qSLWS10-1	10	4.533	5.035	3	2.59–2.59	5.80–5.80

**FIGURE 5:**
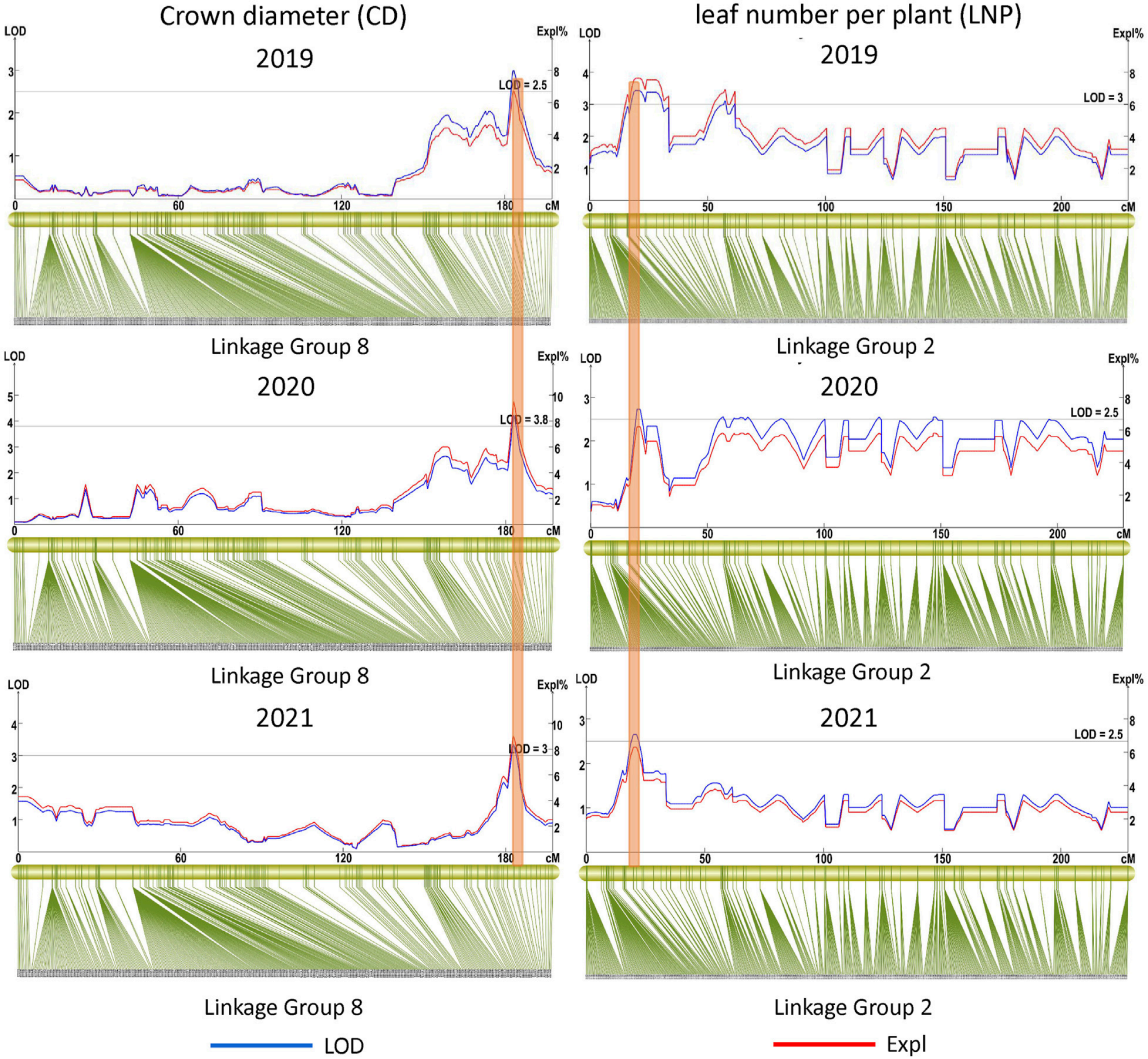
Repeatable QTLs for three dynamic phenotyping time points.

Eighteen QTLs were detected for the six growth traits except PH, including nine for CD, two for BSBN, one for MSH, three for SI, and three for SBD (Table 3). Two major-effect QTLs, *qBSBN7-1* and *qBSBN7-2* (PVE = 10–10.1%), were identified with LOD>4, and *qBSBN7-1* could be detected in both 2020 and 2021 (Table 3). The LOD of *qCD4-1* in 2019 was slightly over 2.5 but was supported by 31 SNPs. Notably, *qCD8*, which was located in the 182.843–184.973 cM interval, could be repeatably mapped over 3 years, indicating that this QTL could be expressed continuously, contributing to the establishment of the crown (Figure 5). Especially in 2020, the LOD value of *qCD8* exceeded four, and PVE exceeded 9%. For SI, there were two QTLs (*qSI4-1* and *qSI4-2*) with a spacing of only 1.322 cM, which were supported by 16 and 12 SNPs, respectively.

In the QTLs of the leaf-related phenotype, four QTLs for LLLS were mapped to LG4, LG9, LG10 and LG11, of which the highest LOD value of *qLLLS9* in 2020 was up to 5.3, and the corresponding PVE was equal to 11.7%. The largest number of identified QTLs belonged to trait LNP, up to 12, and was primarily distributed on LG10, of which *qLNP2-1*, located at 19.951–28.282 cM, was detected for three consecutive years (Table 3; Figure 5). *qLNP2-2* and *qLNP2-10* were 0.502 cM apart, implying that they might be the same QTL. A total of five QTLs were responsible for SLLS, of which *qSLLS11-1*, *qSLLS11-2* and *qSLLS11-3* gathered between 211.278 and 223.331 cM, with 41 supported SNPs. There were three QTLs for LNS on LG7, LG8 and LG9. With respect to SLWS and LLWS, three and two QTLs were mapped, respectively.

**FIGURE 6:**
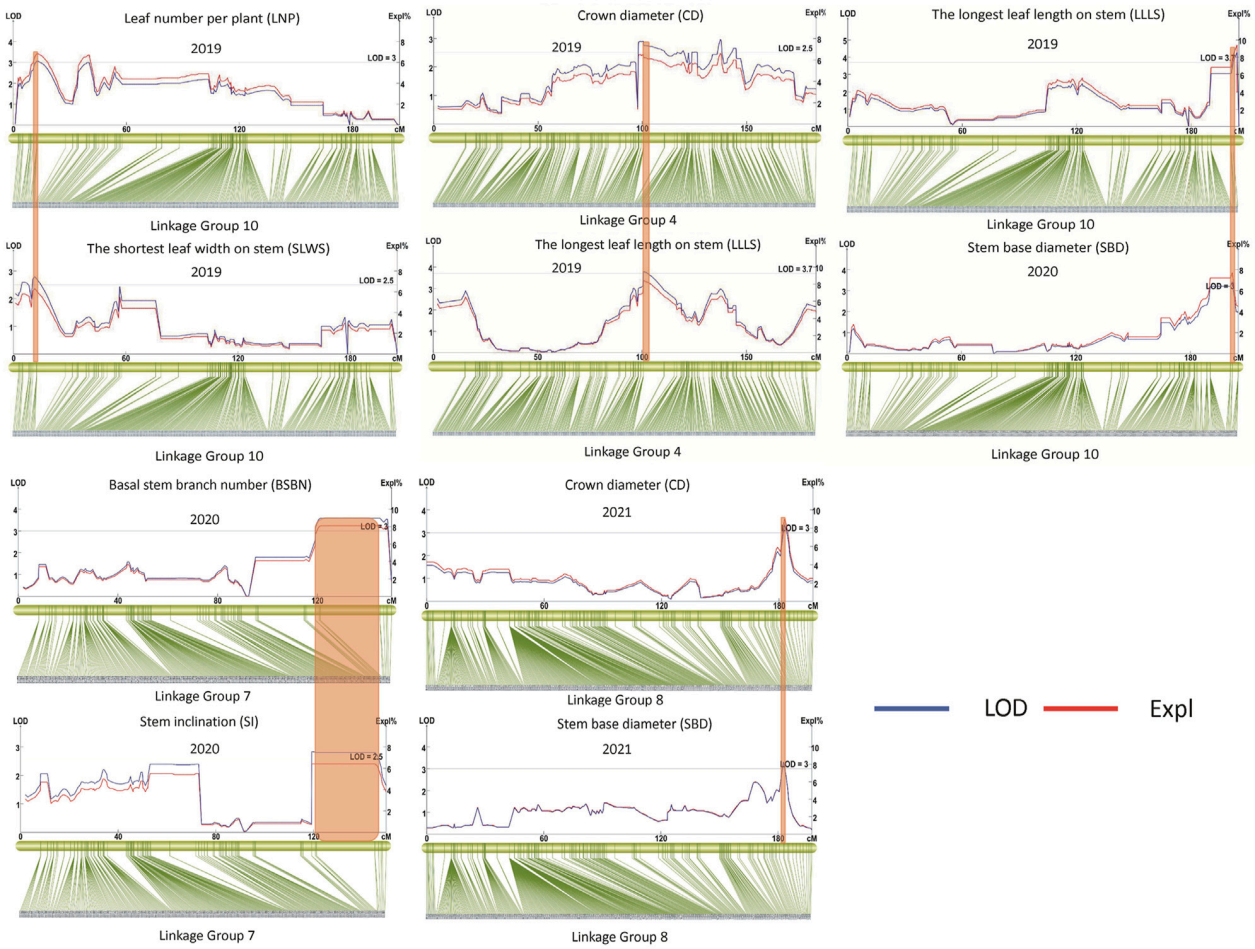
Potential pleiotropism QTLs.

We continued to explore the QTLs among different phenotypes and found that there were five pairs of QTLs with shared regions, including *qCD8* and *qSBD8*, *qBSBN7* and *qSI7*, *qCD4-1* and *qLLLS4*, *qLNP10* and *qSLWS10-2*, *qSBD10* and *qLLLS10* (Table 3; Figure 6), suggesting that each pair underlying a single QTL and pleiotropism might play a significant role in gardenia morphogenesis and vegetative development. The structural and functional gene annotations of the above stable and potential pleiotropism QTLs were isolated, resulting in 2,514 nonredundant genes and the corresponding annotation information (Supplementary Table S6–S7).

### KASP-Based SNP Confirmation

We selected 17 SNP-based KASP markers on chromosomes 2, 7, 9, and 11 and a total of 96 samples for SNP accuracy verification. There were 15 out of 17 markers with successful fluorescence signals in the HC KASP platform, accounting for 88.24%. Among these 15 markers, 13 SNP markers showed genotypes consistent with the GBS results of each individual (Supplementary Table S8), indicating the accuracy of the sequencing analysis.

### Synteny Analyses

**FIGURE 7:**
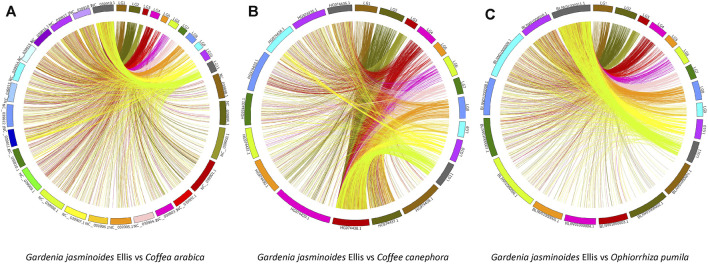
Synteny analyses between the genetic map of gardenia and the genomes of *Coffea arabica* **(A)**, *Coffea canephora* **(B)**, and *Ophiorrhiza pumila* **(C)**.

We used this high-density genetic map to investigate the evolutionary relationship of Rubiaceae species, as shown in Figure 7. Different levels of synteny were observed between the LGs of gardenia between *C. arabica* (A), *C. canephora* (B) and *O. pumila* (C). Specifically, relatively strong synteny was consistently noted between LG11 of gardenia and NC_039,919.1 of *C. arabica*, HG974438.1 of *C. canephora*, and Bliw01000011.1 of *O. pumila*, indicating that LG11 was more conserved than other LGs. In addition, this type of stronger collinearity was also found between these pairs: the pair LG10 and *C. arabica*’s chromosome NC_039,919.1, the pair LG4 and *C. canephora*’s chromosome HG974438.1, and the pair LG10 and *O. pumila*’s chromosome BLIW01000011.1. Moreover, chromosomes NC_039,917.1, NC_039,918.1 and NC_039,919.1 demonstrated higher collinearity than other chromosomes in *C. arabica*. Similarly, chromosomes HG974438.1, HG974437.1 and *HG974436.1* in *C. canephora* and chromosomes BLIW01000010.1 and BLIW01000011.1 in *O. pumila* displayed stronger synteny than other corresponding chromosomes.

## Discussion

Gardenia is a type of gardening species that has medicinal and industrial value. At present, there is less genetic research on this species. In this study, after constructing an F_1_ segregating population, the first high-density genetic map of gardenia was accomplished using a high-throughput sequencing method.

Three-year dynamic QTL positioning identified a panel of vegetative growth-related QTLs. We believe that this research will open a new avenue for gardenia molecular genetic research.

NGS has greatly accelerated the process of QTL mapping according to forward genetics, as primarily performed by bulk segregation analysis (BSA), high-density genetic map-based QTL mapping and genome-wide association studies (GWASs) in horticultural plants (Ban and Xu, 2020; Ferrão et al., 2020; Song et al., 2020). GBS, which is rooted in NGS, has opened up new possibilities for genome-wide SNP mining without high investments (Chung et al., 2017). At the cost of approximately 1.8 Gb sequencing data per sample, 154,909 dual-calling SNPs were *de novo* developed in the present study. Hereafter, the sequencing depth, segregation distortion and integrity control were processed to guarantee a high-quality panel of SNPs for high-density genetic map construction. Using an F_1_ population of 200 plants of gardenia and GBS-based genotyping, a high-density genetic map harboring 4,249 SNPs was constructed, which showed high resolution (0.46 cM per adjacent SNPs) and satisfied marker orders with an approximately 0.8 collinearity compared with the reference genome, a similar standard as in other species (Ji et al., 2018; Lu et al., 2019; Wang et al., 2020). To evaluate the SNP accuracy, we selected 17 SNPs and genotyped 96 F_1_ individuals, and high consistency between the KASP and GBS genotypes was observed according to Song’s report (Song et al., 2020). These results indicated that a high-quality and high-density genetic map was generated. Furthermore, the genetic map was used for the QTL mapping responsible for early growth and development traits for three dynamic years, and a total of 49 QTLs for 12 traits (CD, BSBN, SI, PH, MSH, SBD, LNS, LNP, LLLS, LLWS, SLLS, and SLWS) were identified. Because the most useful organ was the gardenia fruit, further QTLs associated with fruit-related traits, such as the weight, shape, size or functional substance content, could be expected in 2022 or later, when all F_1_ gardenia individuals will transfer to reproductive growth. This high-density genetic map might provide a new lesson for molecular genetic research in gardenia.

As mentioned above, 49 QTLs for 12 traits were mapped, including three stably expressed QTLs, *qBSBN7-1*, *qCD8* and *qLNP2-1* (Table 3). These QTLs played persistent roles in the corresponding morphogenesis during the vegetative period and were valuable and useful for MAS-based breeding programs (Jamshed et al., 2016; Galiano-Carneiro et al., 2021). In addition, we also found five regions within two QTLs (*qCD8* and *qSBD8*, *qBSBN7* and *qSI7*, *qCD4-1* and *qLLLS4*, *qLNP10* and *qSLWS10-2*, *qSBD10* and *qLLLS10*), suggesting that each region underlies a single QTL with pleiotropism (Sun et al., 2017; Wang et al., 2018). Notably, there was a genetic basis for the phenotypic correlation between CD and SBD, and CD and LLLS, which was consistent with the strong correlations between CD and SBD (0.40***), and CD and LLLS (0.53***) (Figure 2). The SNPs underlying these stable and pleiotropic QTLs could be further converted into KASP markers and potentially used as MAS markers. Further gene cloning may also benefit from the gene structural and functional annotations underlying stable and potential pleiotropism QTLs (Supplementary Table S6, S7).

Generally, gardenia plants are focused on vegetative development for the first 3 years after seed germination, which is called the juvenile period. A gradual declining trend in QTL numbers was observed from 2019, 2020 to 2021, which might be associated with the fact that the channels of vegetative development gradually slowed, while reproductive growth gradually opened. Furthermore, some year-specific QTLs were found except stable and pleiotropism potential QTLs, which could be explained by specific functions in plant growth phases. This phenotyping-derived dynamic QTL mapping based on continuous development time points has recently been performed in peaches (Desnoues et al., 2016), *Populus* (Du et al., 2019), *Catalpa bungei* (Lu et al., 2019), and chrysanthemum (Ao et al., 2019). Compared with these studies, one shortcoming of this study is that phenotyping was conducted at only three time points, which might limit the dissection resolution of the developmental trait inheritance. Currently, high-throughput phenomics combining spectral imaging and machine learning methods provides particular insight into the deciphering of dynamic phenotypes in a way that is plant damage-free (Li and Sillanpää 2015; Adams et al., 2020; Streich et al., 2020; Zhu et al., 2021). Novel dynamic QTL perspectives might be enabled by employing phenomic methods in the near future.

The complex synteny of gardenia with *C. arabica*, *C. canephora* and *O. pumila* (Figure 7) implied that widely chromosomal fission and fusion have happened after their divergence from the common ancestor, similar to other species (Luo et al., 2020; Yang et al., 2020). The synteny levels of all LGs in gardenia indicated that different chromosomes underwent different evolutionary process. Strong synteny in LG10, LG11 and LG4 of gardenia demonstrated that these LGs were more conserved than other LGs. The sequences underlying these conserved regions could be potentially used to speculate the corresponding genetic information of species in *Salicaceae*, and the QTLs on LG4, LG10 and LG11 (*qCD4-1*, *qSBD10*, *qSLLS11-3*, etc.), potentially orthologous QTLs (Rinaldi et al., 2016; Webb et al., 2016), could be applied for comparative mapping in other species of *Rubiaceae*. Ta°Cken together, the synteny analyses of this paper may lay a foundation for subsequent comparative genomic research.

One ultimate goal of QTL mapping is to perform QTL fine mapping, screening and gene cloning of candidate genes. This goal was commonly achieved in therophyte plants, such as through map-based cloning (Jia et al., 2020; Yu et al., 2020; Sierra-Orozco et al., 2021). However, for many perennial species, it takes more than 6 years to perform hybridization and further backcrossing, and the population size is always restricted to several hundred, which leads to limited recombination. This limitation makes the process of QTL cloning in perennial species slow. To expedite this process, transcriptomics analysis can be used to call RNA variants and differentially expressed genes (DEGs) within QTL regions (Park et al., 2019; Wen et al., 2019). Other omics analyses, such as metabolomics and proteomics, can also provide useful information on the metabolic chemicals or proteins related to phenotypic variations (Szymański et al., 2020; Mou et al., 2021). In future experimental designs, these analyses will be considered to understand the basis of phenotypic variation comprehensively.

## 5 Conclusion

In this study, we developed a panel of genome-wide high-quality SNPs using the GBS method and providing the first high-density genetic map in the gardenia. SNPs and genetic maps could be useful for further genetic study and evolutionary genomics. Based on this high-density genetic map, 18 and 31 QTLs were identified for growth traits and leaf-related traits at three dynamic phenotyping time points, respectively. Stably expressed QTLs and potential pleiotropism QTLs could be targets for MAS breeding and for further gene cloning.

## Data Availability

The datasets presented in this study can be found in online repositories. The names of the repository/repositories and accession number(s) can be found in the article/Supplementary Material.

## Abbreviations

SNPs, single-nucleotide polymorphisms; QTLs, quantitative trait loci; LOD, logarithm of odds; MAS, marker-assisted selection; NGS, next-generation sequencing; GBS, genotyping-by-sequencing; CD, crown diameter; BSBN, basal stem branch number; SI, stem inclination; PH, plant height; MSH, main stem height; SBD, stem base diameter; LNS, leaf number on stem; LNP, leaf number per plant; LLLS, longest leaf length on stem; LLWS, longest leaf width on stem; SLLS, shortest leaf length on stem; SLWS, shortest leaf width on stem; CV, coefficient of variation; LGs, linkage groups; PVE, phenotypic variance explained; KASP, kompetitive allele-specific PCR; CP, cross pollination; DEGs, differentially expressed genes.
