# Supplementary figures and images for: A High-Density Genetic Map Enables Genome Synteny and QTL Mapping of Vegetative Growth and Leaf Traits in Gardenia
Source: Front Genet. 2022 Jan 4;12:802738. doi: 10.3389/fgene.2021.802738 (PMC8817757; doi:10.3389/fgene.2021.802738)

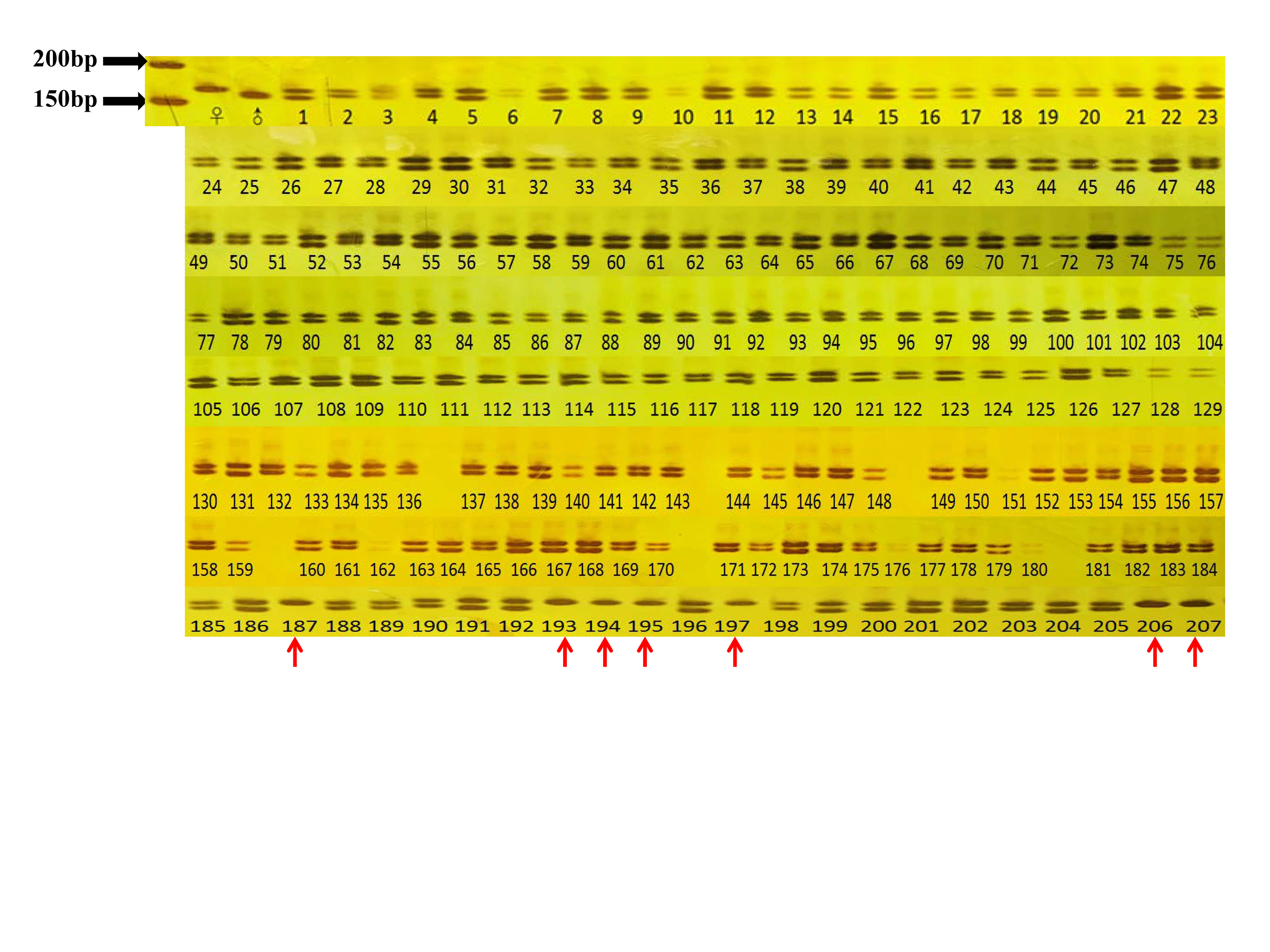

Supplement: Supplementary file 3 [file Image1.JPEG]

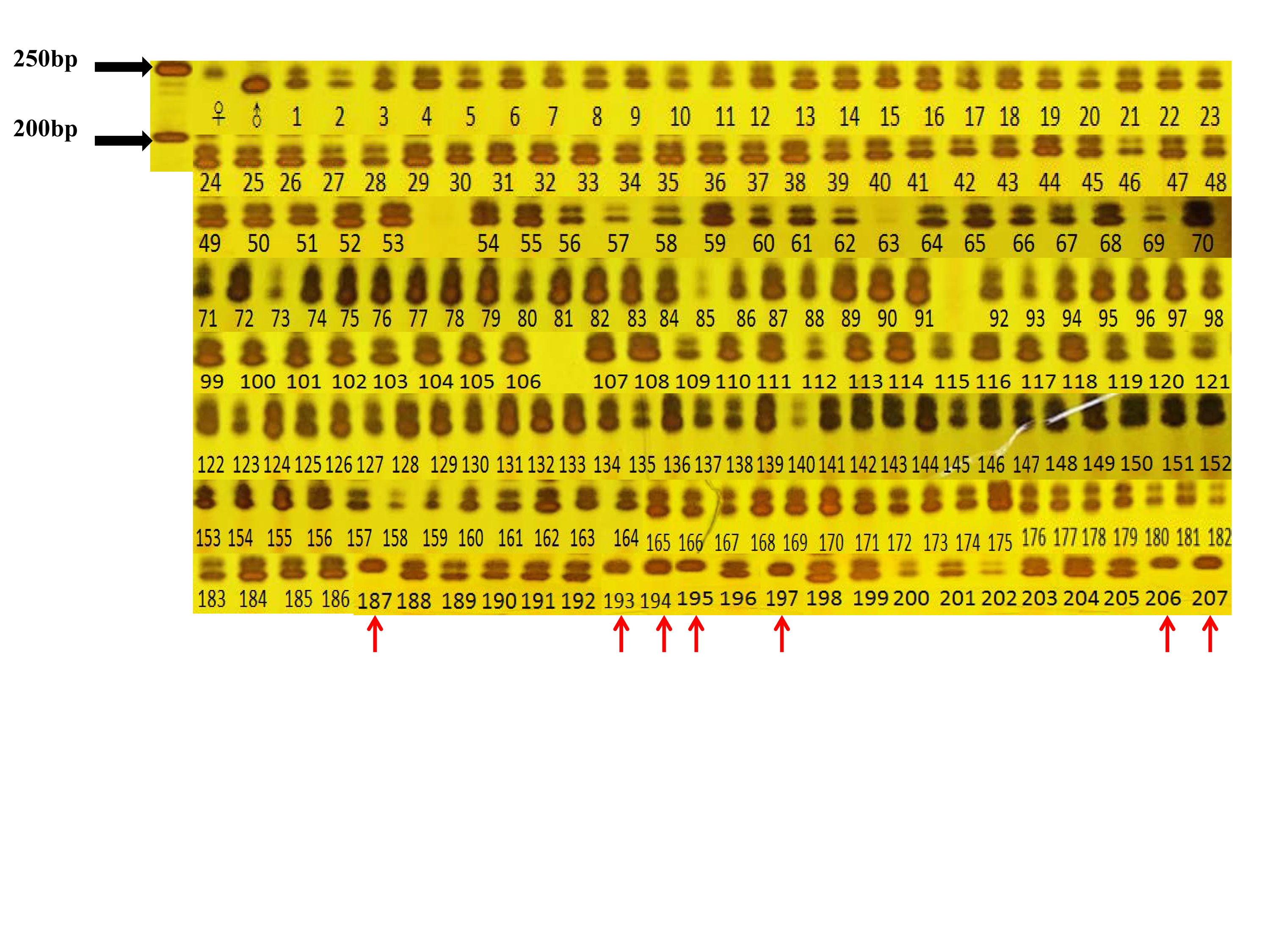

Supplement: Supplementary file 4 [file Image2.JPEG]
