# Supplementary material for: A High-Density Genetic Map Enables Genome Synteny and QTL Mapping of Vegetative Growth and Leaf Traits in Gardenia
Source: Front Genet. 2022 Jan 4;12:802738. doi: 10.3389/fgene.2021.802738 (PMC8817757; doi:10.3389/fgene.2021.802738)
Supplement: Supplementary file 5 [file Table9.DOCX]

Supplementary Material

## Supplementary Figures

**Supplementary Figure S1.** Polyacrylamide gel electrophoresis results using SSR (eGJ026). ♀: GD1 indicates female parent;♂: AX5 indicates male parent; 1-207:207 F_1_ individuals; Red arrow indicates non-hybrid individual.

**Supplementary Figure S2.** Polyacrylamide gel electrophoresis results using SSR (eGJ118). ♀: GD1 indicates female parent;♂: AX5 indicates male parent; 1-207:207 F_1_ individuals; Red arrow indicates non-hybrid individual.

## Supplementary Tables

**Supplementary Table S1.** Primers for KASP validation.

**Supplementary Table S2.** Data Summary for phenotype.

**Supplementary Table S3.** Sequencing data statistics.

**Supplementary Table S4**. Genetic and physical positions of SNP markers on the genetic map.

**Supplementary Table S5.** Linkage group collinearity spearman coefficient.

**Supplementary Table S6**. Structral annotations of stable QTL regions.

**Supplementary Table S7**. Gene functional annotations of stable QTL regions.

**Supplementary Table S8.** KASP genotyping results.
